# Supplementary material for: Soil communities following harvest have different early successional dynamics compared with post-wildfire patterns
Source: Sci Rep. 2025 Dec 4;15:43413. doi: 10.1038/s41598-025-16373-y (PMC12689615; doi:10.1038/s41598-025-16373-y)
Supplement: Supplementary file 1 — Supplementary Material 1 [file 41598_2025_16373_MOESM1_ESM.docx]

**Supplementary material**

**Soil communities following harvest have different early successional dynamics compared with post-wildfire patterns**

Teresita M. Porter, Dave M. Morris, Emily Smenderovac, Erik J.S. Emilson, Lisa Venier

**Supplementary Results**

**Figure S1. Rarefaction curves plateau indicating sequencing depth was sufficient for each amplicon.** Across all samples in this study, the number of sequence variants (SVs) detected from bacteria (16S) is highest, followed by fungi (ITS), and least for arthropods (COI).

**Figure S2. Overview of taxa from wildfire, full tree clearcut, and salvage harvest sites.** Sequence variant richness (total number of unique SVs) is shown for (a) bacteria, (b) fungi, and (c) arthropods showing dominant taxa for each disturbance type at each stand development stage. The same dominant classes were also detected with relative read abundance (data not shown). Abbreviations: establishment (E), crown closure (CC), early self-thinning (EST), late self-thinning (LST), mature (M).

**Table S1. Overview of site characteristics for each disturbance type and stand development stage.**

| **Disturbance type** | **Site characteristic** | **Stand Establishment** | **Crown Closure** | **Early Self-Thinning** | **Late Self-Thinning** | **Mature** |
| --- | --- | --- | --- | --- | --- | --- |
| **Wildfire** | **Dominant understory** | Moss, deadwood (fire origin), blueberry | Moss, deadwood (fire origin), bunchberry | Moss, litter | Moss, deadwood (self-thinning origin), mayflower | Moss, lichen, honey suckle |
|  | **pH**  **Bryophyte**  **Organic**  **Mineral**  **Pooled layers** | 5.68 ± 0.12  5.88 ± 0.19  5.73 ± 0.30  5.76 ± 0.23 | 5.19 ± 0.52  4.96 ± 0.48  5.43 ± 0.33  5.19 ± 0.48 | 5.00 ± 0.35  4.62 ± 0.09  5.51 ± 0.33  5.04 ± 0.46 | 5.17 ± 0.44  4.69 ± 0.12  5.32 ± 0.13  5.06 ± 0.38 | 5.12 ± 0.23  4.55 ± 0.20  5.23 ± 0.16  4.97 ± 0.36 |
|  | **TOC (g/kg)**  **Bryophyte**  **Organic**  **Mineral**  **Pooled layers** | 432 ± 3.57  288 ± 46.9  17.4 ± 0.75  244 ± 180 | 500 ± 15.4  485 ± 22.0  12.3 ± 2.73  333 ± 231 | 494 ± 9.32  486 ± 9.31  9.55 ± 0.241  330 ± 231 | 502 ± 6.30  491 ± 30.1  16.0 ± 4.47  336 ± 234 | 490 ± 14.4  467 ± 15.5  10.8 ± 3.29  323 ± 225 |
|  | **TN (g/kg)**  **Bryophyte**  **Organic**  **Mineral**  **Pooled layers** | 12.9 ± 0.93  12.4 ± 1.53  0.89 ± 0.06  8.58 ± 5.79 | 12.3 ± 4.83  14.4 ± 4.86  0.5 ± 0.03  9.07 ± 7.30 | 9.5 ± 1.23  13.1 ± 1.40  0.39 ± 0.06  7.65 ± 5.54 | 11.2 ± 1.05  14.2 ± 0.97  0.68 ± 0.15  8.67 ± 6.01 | 9.9 ± 0.97  13.1 ± 1.01  0.48 ± 0.18  7.81 ± 5.50 |
| **Full tree clearcut harvest** | **Dominant understory** | Blueberry, Honeysuckle, Moss, Bunchberry, Raspberry | Moss, Litter, Labrador Tea, Bunchberry, Blue-bead Lily, Blueberry | Moss, Litter, Bunchberry | Moss, Bunchberry, deadwood (self-thinning) | - |
|  | **pH**  **Bryophyte**  **Organic**  **Mineral**  **Pooled layers** | 5.34 ± 0.39  5.09 ± 0.55  5.31 ± 0.21  5.24 ± 0.41 | 5.42 ± 0.12  5.41 ± 0.36  5.46 ± 0.28  5.43 ± 0.26 | 4.78 ± 0.83  4.47 ± 0.69  5.22 ± 0.14  4.82 ± 0.68 | 5.18 ± 0.14  5.06 ± 0.05  5.52 ± 0.19  5.26 ± 0.24 | - |
|  | **TOC (g/kg)**  **Bryophyte**  **Organic**  **Mineral**  **Pooled layers** | 474 ± 10.9  428 ± 53.9  14.1 ± 0.54  306 ± 213 | 499 ± 14.2  477 ± 14.3  13.3 ± 1.58  330 ± 229 | 504 ± 10.8  485 ± 13.1  13.8 ± 4.62  334 ± 231 | 490 ± 3.36  478 ± 27.0  11.4 ± 5.15  326 ± 230 | - |
|  | **TN (g/kg)**  **Bryophyte**  **Organic**  **Mineral**  **Pooled layers** | 10.8 ± 1.46  13.6 ± 1.84  0.63 ± 0.06  8.35 ± 5.83 | 12.1 ± 0.46  16.3 ± 0.71  0.61 ± 0.15  9.67 ± 6.78 | 12.6 ± 0.45  16.5 ± 0.54  0.55 ± 0.15  9.89 ± 6.94 | 10.9 ± 0.14  14.2 ± 1.82  0.42 ± 0.16  8.52 ± 6.14 | - |
| **Salvage harvest** | **Dominant understory** | Litter, Blueberry | Lichen | - | - | - |
|  | **pH**  **Bryophyte**  **Organic**  **Mineral**  **Pooled layers** | 5.39 ± 0.14  5.50 ± 0.13  5.82 ± 0.09  5.56 ± 0.22 | 4.89 ± 0.17  4.68 ± 0.20  5.46 ± 0.05  5.01 ± 0.37 | - | - | - |
|  | **TOC (g/kg)**  **Bryophyte**  **Organic**  **Mineral**  **Pooled layers** | 470 ± 22.6  412 ± 25.5  15.4 ± 7.39  310 ± 203 | 473 ± 3.48  423 ± 43.9  8.3 ± 1.68  301 ± 214 | - | - | - |
|  | **TN (g/kg)**  **Bryophyte**  **Organic**  **Mineral**  **Pooled layers** | 9.53 ± 1.89  10.8 ± 1.78  0.63 ± 0.24  7.24 ± 4.76 | 9.73 ± 1.08  12.0 ± 1.09  0.34 ± 0.06  7.34 ± 5.20 | - | - | - |

Note: Extractable phosphorus was also measured but not included here because it was not significantly correlated with soil community shifts, neither in this study nor in our previous study focusing on succession in post-wildfire soils (Porter et al. 2023).

Abbreviations: Not sampled (-), standard deviation (sd), total nitrogen (TN), total organic carbon (TOC).

**Table S2: Reads and sequence variants (SVs) at each major bioinformatic step.**

| **Step** | **COI-BE** | **COI-F230** | **ITS2** | **16Sv4v5** | **Total** |
| --- | --- | --- | --- | --- | --- |
| **Raw reads** | 7,726,307 x 2 | 18,578,038 x 2 | 11,376,813 x 2 | 12,039,996 x 2 | 49,721,154 x 2 |
| **Paired reads** | 6,971,930 | 17,041,560 | 9,780,624 | 10,411,151 | 44,205,265 |
| **Primer-trimmed reads** | 6,014,076 | 16,650,830 | 9,567,562 | 10,208,799 | 42,441,267 |
| **Denoised SVs^a^ (Reads)** | 1,260  (97,266) | 7,733 (2,741,702) | 27,884 (3,952,018) | 78,091 (3,286,199) | 114,968 (10,077,185) |

^a^ Removal of sequence clusters with less than 3 reads, sequences with errors, putative chimeras, and obvious pseudogenes to create a set of sequence variants (SVs).

**References**

Porter, Teresita M, Emily Smenderovac, Dave Morris, and Lisa Venier. 2023. “All Boreal Forest Successional Stages Needed to Maintain the Full Suite of Soil Biodiversity, Community Composition, and Function Following Wildfire.” *Scientific Reports* 13:7978.
